# Under-attribution in self-agency on pre-reflexive task connected to positive schizotypal traits among healthy students

**DOI:** 10.1192/j.eurpsy.2024.1394

**Published:** 2024-08-27

**Authors:** I. Szendi, N. Domján, H. Pásztor, T. Jenei, O. Bóna, C. Kovács, A. Pejin, P. Pajkossy, Á. Szőllősi, M. Racsmány

**Affiliations:** ^1^Department of Personality, Clinical and Health Psychology, University of Szeged, Szeged; ^2^Department of Psychiatry, Kiskunhalas Semmelweis Hospital, Kiskunhalas; ^3^Department of Psychiatry, University of Szeged, Szeged; ^4^Department of Cognitive Science, Budapest University of Technology and Economics, Budapest; ^5^Department of Cognitive and Neuropsychology, University of Szeged, Szeged, Hungary

## Abstract

**Introduction:**

The aim of this study was to identify low-risk traits of schizophrenia among healthy undergraduate student volunteers, and the investigation of these traits with regards to their specificity in contrast to individuals with a latent disposition towards bipolar disorder. Self-agency, as a phenomenon closely related to psychomotor functioning, provides a unique opportunity for the investigation of subjective self-perception.

**Objectives:**

The implicit self-agency performances that are considered illness- (or risk state-) specific were compared between groups to find early markers of a specific schizotypic developmental path.

**Methods:**

In a sample of 710 healthy university students, with the help of screening questionnaires, we were able to successfully form two risk groups, in one of them the emphasis on cyclothymia (CTF: Cyclothymia factor group, N=25), and in the other (PSF: Positive schizotypy factor group, N=26) the tendency to unusual experiences and paranoid thinking emphasis was typical. We assigned a properly matched control group (N=29) displaying both features on average. We focused on the implicit aspect of self-agency, using the well-known paradigm of intentional binding, as well as the self-developed device that exclusively tests the pre-reflexive feeling of movement initiation, the sense of self-agency.

**Results:**

During the examination of intentional binding, although the specific predictive and retrospective component indicators did not show any significant difference for either group, the association of the sound alone could induce a binding effect in the control group. In the predictable frequency condition, there was a strong significant effect (W = 65.00, p = .007, rrb = -.60), and in the non-predictable condition a trend-level effect. Remarkably, this binding effect did not develop in either the CTF or PSF groups, indicating an implicit agency impairment in both risk groups. However, during the examination of sense of self-agency, we observed a disturbance specifically among healthy college students with positive schizotypal traits, in the form of falsely attributing their movement initiation to external influences. The percentage of this ‘miss’-type answering differed between groups, H(2) = 7.68, p = .021, ε2 = .10. The Dwass-SteelCritchlow-Fligner pairwise comparisons showed that this difference was due to the PSF Group showing a significant difference from the Control Group (W = -3.83, p = .019), but not from the CTF group, and the CTF Group also did not differ from the Control Group.

**Conclusions:**

Thus, in premorbid conditions, in at-risk groups of non-help-seeking individuals, or in cases of early detection of prodromal abnormalities, objective confirmation of suspected susceptibility to schizophrenia may be aided by, among other things, instrumental assessment of self-agency.

**Disclosure of Interest:**

None Declared

